# Intra-aortic balloon pump support and early mortality after surgical repair of post-infarction ventricular septal rupture: A 24-year real-world cohort

**DOI:** 10.1016/j.ahjo.2026.100863

**Published:** 2026-08-20

**Authors:** Italo Menezes Ferreira, Mariana Ayaka Yamashita, Marina Carvalho Giannini, Gabriel Martins Pistori, Vinicius de Amorim e Jorge, Alex Cesar Lacuna, Laíz Teixeira Pontes, Raphael Paris Rosan, Antônio Carlos Mugayar Bianco, Mario Issa, Kleber Franchini, Fausto Feres, Louis Nakayama Ohe

**Affiliations:** a Cardiac Intensive Care Unit, Instituto Dante Pazzanese de Cardiologia, São Paulo, Brazil; b Department of Interventional Cardiology, Instituto Dante Pazzanese de Cardiologia, São Paulo, Brazil; c Emergency Department, Instituto Dante Pazzanese de Cardiologia, São Paulo, Brazil; d Department of Cardiovascular Surgery, Instituto Dante Pazzanese de Cardiologia, São Paulo, Brazil; e Department of Research, Instituto Dante Pazzanese de Cardiologia, São Paulo, Brazil

**Keywords:** Ventricular septal rupture, Acute myocardial infarction, Intra-aortic balloon pump, Mechanical circulatory support, Cardiogenic shock

## Abstract

**Background:**

Post-infarction ventricular septal rupture (VSR) remains a rare but highly lethal complication of acute myocardial infarction, with persistently high early mortality. Although surgical repair is the definitive treatment, the optimal perioperative management strategy remains uncertain, particularly regarding the role of intra-aortic balloon pump (IABP) support.

**Methods:**

We conducted a retrospective cohort study including consecutive patients with post-infarction VSR who underwent surgical repair at a tertiary cardiovascular centre over a 24-year period. Clinical characteristics, timing of diagnosis and surgery, use and timing of IABP support, and 30-day all-cause mortality were analysed. Multivariable logistic regression was performed to identify independent predictors of early mortality. Patients managed conservatively or treated exclusively with percutaneous closure were not included.

**Results:**

A total of 59 patients were included (mean age 65.3 ± 10.2 years; 57.6% male). Overall 30-day mortality was 54.4%. IABP support was used in 64.4% of patients, including 33.9% pre-operatively and 30.5% post-operatively. Non-survivors experienced longer delays from symptom onset to surgery and from hospital admission to surgery. In multivariable analysis, post-operative IABP use was independently associated with lower 30-day mortality (OR 0.014, 95% CI 0.001–0.120; *p* = 0.001). Pre-operative IABP use showed a protective trend but did not reach statistical significance (OR 0.161, 95% CI 0.016–1.062; *p* = 0.077).

**Conclusions:**

In this real-world cohort characterised by delayed diagnosis and referral, perioperative IABP support was strongly associated with improved early survival after surgical repair of post-infarction VSR, supporting its pragmatic role as haemodynamic support in selected patients.

## Highlights

• Post-infarction ventricular septal rupture remains highly lethal.
• Delayed diagnosis and referral were frequent in this real-world cohort.
• Post-operative IABP use emerged as an independent predictor of survival.
• IABP may represent a pragmatic bridge-to-repair strategy in VSR.

## Graphical abstract

Post-operative intra-aortic balloon pump support was associated with reduced 30-day mortality after surgical repair of post-infarction ventricular septal rupture.

**Figure:**
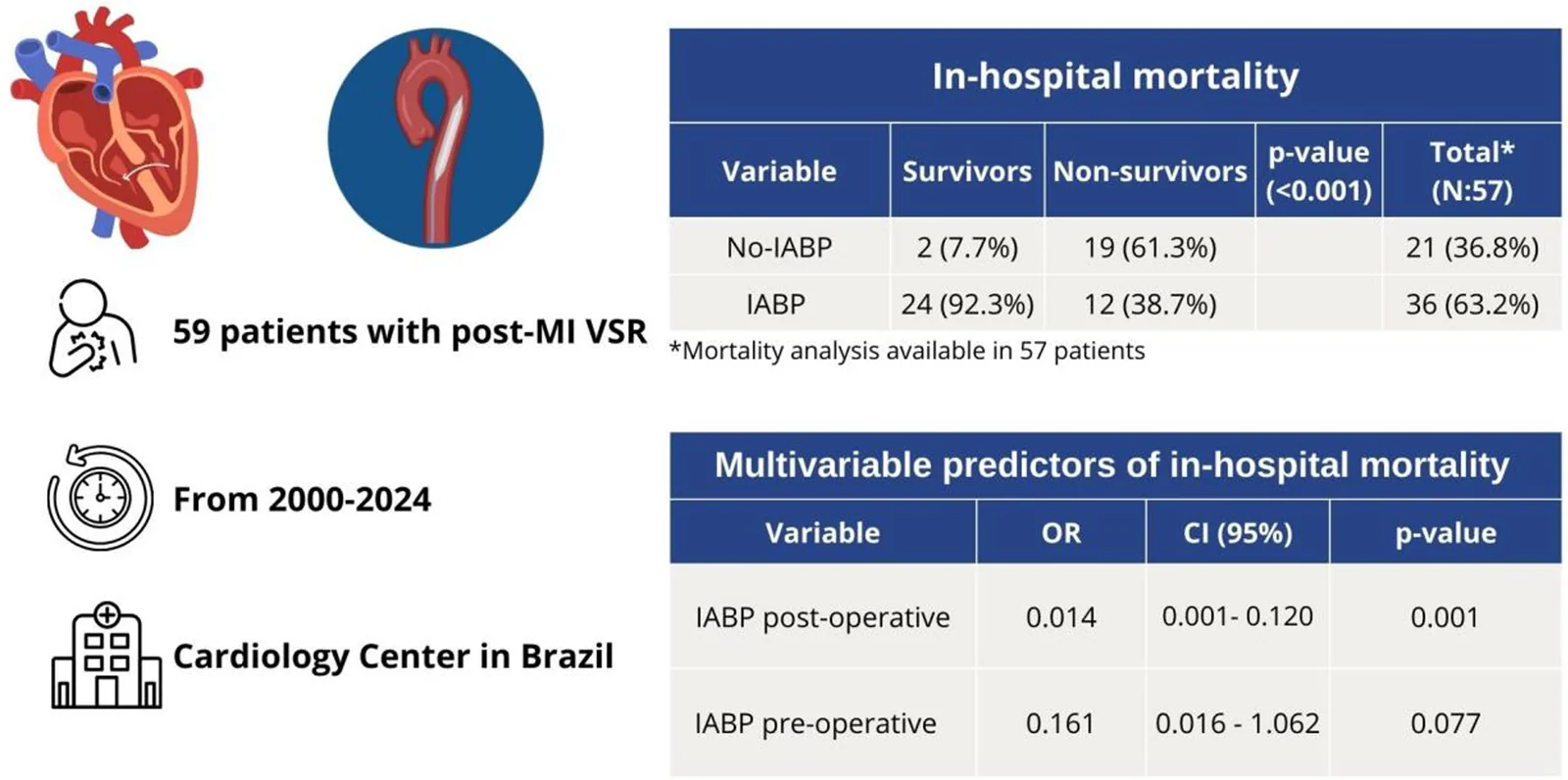

## 1 Introduction

Cardiovascular disease remains the leading cause of death worldwide, with ischaemic heart disease representing its most common acute presentation [1]. Despite substantial advances in early reperfusion strategies, acute myocardial infarction (AMI) continues to be associated with a significant burden of severe complications, particularly cardiogenic shock and mechanical complications, both of which carry unacceptably high mortality rates [2].

Mechanical complications of AMI—including left ventricular free wall rupture, acute severe mitral regurgitation secondary to papillary muscle rupture, and ventricular septal rupture (VSR)—represent catastrophic events [3]. Although their incidence has markedly declined in the contemporary reperfusion era, currently reported between approximately 0.12% and 0.44%, these complications remain associated with haemodynamic collapse and cardiogenic shock in more than half of affected patients [4], [5].

Among these entities, post-infarction VSR remains one of the most challenging conditions in acute cardiovascular care. Surgical repair is considered the definitive treatment; however, the optimal timing of intervention remains controversial. Early surgery is often mandated by refractory haemodynamic instability but is associated with high operative mortality due to friable, necrotic myocardial tissue. Conversely, delayed repair allows scar formation and tissue consolidation, facilitating surgical closure, but exposes patients to prolonged periods of haemodynamic compromise [5].

In this complex clinical context, mechanical circulatory support (MCS) plays a pivotal role as a bridge to definitive repair. The intra-aortic balloon pump (IABP), once a cornerstone of haemodynamic support in AMI, has experienced a substantial decline in use following neutral outcomes in unselected cardiogenic shock populations. However, its physiological effects—afterload reduction, augmentation of coronary perfusion, and left ventricular unloading—remain particularly relevant in patients with mechanical complications, especially VSR, in whom shunt reduction and haemodynamic stabilisation are critical [4], [5].

The present study aimed to evaluate the contemporary role of intra-aortic balloon pump support in patients with post-infarction ventricular septal rupture, focusing on its association with 30-day mortality and its interaction with surgical timing, using data from a 24-year retrospective cohort at a tertiary cardiovascular centre.

## 2 Methods

### 2.1 Study design, data collection, and outcomes

This retrospective cohort study included adult patients with post-infarction ventricular septal rupture who underwent surgical repair at a tertiary cardiovascular referral centre over a 24-year period. Patients managed conservatively or treated exclusively with percutaneous closure were not included.

Demographic characteristics, cardiovascular risk factors, laboratory findings, echocardiographic parameters, coronary anatomy, use and timing of intra-aortic balloon pump support, surgical timing, and clinical outcomes were retrospectively extracted from medical records. Particular attention was given to time intervals between symptom onset, hospital admission, and surgical repair.

The primary endpoint was all-cause 30-day mortality. In this cohort, all recorded deaths occurred during the index hospitalisation; therefore, in-hospital mortality and 30-day mortality were numerically identical.

Due to the retrospective design, not all variables were available for all patients. For each analysis, the denominator reflects the number of patients with complete data for the variable or outcome of interest (complete-case analysis). Two patients had incomplete documentation regarding 30-day outcome status and were therefore excluded from mortality analyses. No data imputation was performed, as missingness was likely non-random and imputation could have introduced additional bias in this small, high-risk cohort.

Because the source dataset classified IABP exposure only as no IABP, pre-operative IABP, or post-operative IABP, we could not determine whether patients in the pre-operative and post-operative categories had support exclusively during that phase or in both phases. Accordingly, IABP was analysed as a three-level exposure classification rather than as mutually exclusive time-dependent treatment states.

ECMO was not performed at our institution during the study period and was not part of routine service availability. Referral source, shock severity at the time of surgery, cardiac arrest episodes, and concomitant CABG were not consistently available in analyzable form in the structured dataset and therefore were not included in formal analyses.

The study was approved by the institutional ethics committee, and, given its retrospective design, the requirement for informed consent was waived.

### 2.2 Statistical analysis

Continuous variables are presented as mean ± standard deviation or median with interquartile range, as appropriate. Categorical variables are expressed as absolute numbers and percentages. Comparisons between survivors and non-survivors were performed using the Mann–Whitney *U* test for continuous variables and the chi-square test for categorical variables, as appropriate.

Variables with a *p*-value ≤0.20 in univariable analyses were considered for inclusion in a multivariable binary logistic regression model. A stepwise selection approach was applied to identify independent predictors of 30-day all-cause mortality. All tests were two-sided, and a *p*-value <0.05 was considered statistically significant.

## 3 Results

### 3.1 Baseline characteristics

A total of 59 patients with post-infarction ventricular septal rupture were included. The median age was 67 years, and 57.6% were male. Hypertension was the most prevalent cardiovascular risk factor, followed by dyslipidaemia and diabetes mellitus. Pre-existing atherosclerotic cardiovascular disease was present in nearly three-quarters of patients. Left ventricular ejection fraction at presentation was moderately reduced, with a median value of 41% (IQR 35–47) (Table 1).

**Table 1:** Baseline demographic and clinical characteristics.

Variable		Total N
Median age [IQR] — yr	67.0 [57.5–72.0]	59
Male sex — no. (%)	34 (57.6)	59
Medical history — no. (%)		
Current or former smoking	34 (57.6)	59
Hypertension	42 (71.2)	59
Diabetes mellitus	21 (35.6)	59
Hypercholesterolaemia	30 (50.8)	59
Previous atherosclerotic cardiovascular disease – no. (%)	44 (74.6)	59
Aspirin use at admission — no. (%)	35 (59.3)	59
Serum creatinine at admission, mg/dL — median [IQR]	1.0 [1.0–1.8]	58
Lactate at admission mmol/L - median [IQR]	1.40 [0.85; 2.60]	16
BNP at admission pg/L median [IQR]	4015 [0.00; 13,400]	16
Left ventricular ejection fraction, % — median [IQR]	41 [35–47]	56
Renal replacement therapy — no. (%)	10 (17.2)	58
Intra-aortic balloon pump (IABP) use — no. (%)	38 (64.4)	59
Pre-operative IABP use	20 (33.9)	59
Post-operative IABP use	18 (30.5)	59
IABP no use	21 (35.6)	59
Time from symptom onset to hospital admission, days — median [IQR]	8.0 [1.0–26.2]	54
Time from symptom onset to surgery, days — median [IQR]	15.0 [7.0–37.0]	53
Time from hospital admission to surgery, days — median [IQR]	6.0 [2.0–8.0]	58
Number of affected vessels — no. (%)		
1 vessel	42 (76.4)	55
2 vessels	12 (21.8)	55
3 vessels	1(1.8)	55
Infarct-related artery — no. (%)		
Left anterior descending	34 (62.0)	55
Left circumflex	6 (11.0)	55
Right coronary artery	29 (53.0)	55
VSR location — no. (%)		57
Anterior/apical	32 (56.3)	57
Inferior/posterior	18 (31.5)	57
Indeterminate	7 (12.2)	57

Abbreviations: IABP, intra-aortic balloon pump; OR, odds ratio; CI, confidence interval, VSR: ventricular septal rupture.

Median serum creatinine on admission was 1.00 mg/dL (IQR 1.00–1.80). Renal replacement therapy was required in 17.2% of patients during hospitalisation and was significantly more frequent among survivors than non-survivors (32.0% vs. 6.45%; *p* = 0.017). Most patients had single-vessel coronary artery disease (76.4%), with the left anterior descending artery identified as the most frequent culprit vessel (62%), consistent with the predominance of anterior myocardial infarction in this population. Exploratory retrospective classification of VSR location demonstrated a predominance of anterior/apical defects in the available echocardiographic reports (Table 1).

Among the 59 included patients, outcome data at 30 days were available for 57 patients. Overall 30-day mortality in this subgroup was 54.4% (Table 2).

**Table 2:** Comparison of categorical variables according to 30-day all-cause mortality.

Variable	Total N^a^	Survivors	Non-survivors	*p*-Value
Intra-aortic balloon pump - (IABP)				<0.001
No use	21 (36.8%)	2 (7.7%)	19 (61.3%)	
Use	36 (63.2%)	24 (92.3%)	12 (38.7%)	
Renal replacement therapy				0.017
No use	46 (82.2%)	17 (68.0%)	29 (93.5%)	
Use	10 (17.8%)	8 (32.0%)	2 (6.5%)	

a Mortality data were available for 57 patients for IABP and 56 patients for renal replacement therapy.

### 3.2 Surgical timing and IABP use

The median time from symptom onset to hospital admission was 8.0 days (IQR 1.0–26.2). The median time from symptom onset to surgical repair was 15.0 days (IQR 7.0–37.0). The median interval between hospital admission and surgery was 6.0 days (IQR 2.0–8.0). Overall, two-thirds of patients underwent surgical repair more than 10 days after infarction (Table 3).

**Table 3:** Comparison of time-related variables according to 30-day all-cause mortality.

Variable	Survivors (Median [IQR])	Non-survivors (Median [IQR])	p-Value	Total N^a^
Time from symptom onset to hospital admission (days)	7.5 [0.8–14.2]	11.0 [1.0–46.0]	0.122	53
Time from symptom onset to surgery (days)	11.0 [4.5–19.5]	28.0 [10.0–54.0]	0.015	52
Time from hospital admission to surgery (days)	3.0 [1.0–6.0]	7.0 [5.0–12.5]	0.006	56

a Number of patients with complete data available for each time interval.

When analysed as continuous variables using non-parametric methods, longer delays were observed among non-survivors. The median time from symptom onset to surgical repair was significantly longer in patients who died compared with survivors (28.0 days [IQR 10.0–54.0] vs. 11.0 days [IQR 4.5–19.5]; *p* = 0.015). The median interval between hospital admission and surgery was also longer among non-survivors (7.0 days [IQR 5.0–12.5] vs. 3.0 days [IQR 1.0–6.0]; *p* = 0.006) (Table 3).

### 3.3 Association between timing, IABP use, and mortality

Patients who died experienced significantly longer delays between symptom onset and surgical repair, as well as between hospital admission and surgery. However, when surgical timing was categorised (≤10 vs. >10 days), no independent association with 30-day mortality was observed.

In contrast, intra-aortic balloon pump (IABP) use was strongly associated with survival. Survivors exhibited a markedly higher rate of IABP support compared with non-survivors. In multivariable logistic regression analysis, post-operative IABP use emerged as an independent protective factor against 30-day mortality (OR 0.014, 95% CI 0.001–0.120; *p* = 0.001). Pre-operative IABP use was also associated with a substantial reduction in mortality, demonstrating a consistent protective trend, although it did not reach conventional statistical significance (OR 0.161, 95% CI 0.016–1.062; *p* = 0.077) (Table 4).

**Table 4:** Multivariable logistic regression analysis for 30-day all-cause mortality.

Variable	OR	95% CI	p-value
Post-operative IABP use	0.014	0.001–0.120	0.001
Pre-operative IABP use	0.161	0.016–1.062	0.077

Abbreviations: IABP, intra-aortic balloon pump; OR, odds ratio; CI, confidence interval.

**Fig. 1:**
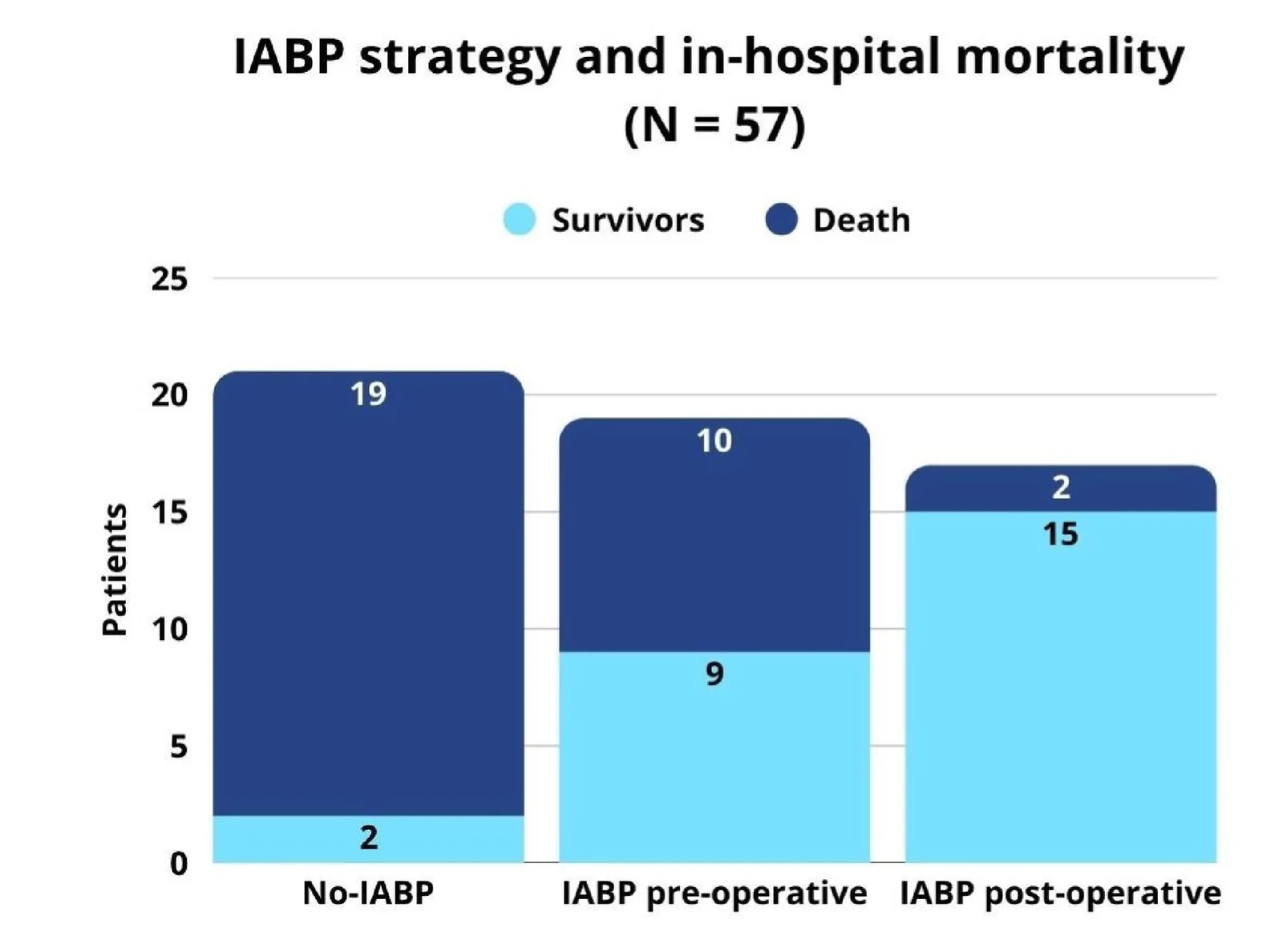
Intra-aortic balloon pump (IABP) strategy and 30-day mortality among patients with post-infarction ventricular septal rupture (*N* = 57).

Overall, IABP support was used in 64.4% of patients, either in the pre-operative or post-operative period (33.9% pre-operatively and 30.5% post-operatively) (Fig. 1).

## 4 Discussion

Ventricular septal rupture remains one of the most lethal mechanical complications of acute myocardial infarction. In the pre-thrombolytic era, its incidence was more than 2% [6]. However, this has markedly declined with the advent of reperfusion therapy. In the GUSTO-I trial, ventricular septal rupture occurred in only 0.2% of patients, reflecting the protective effects of early reperfusion and myocardial salvage—an effect that has been consistently reproduced with the widespread adoption of primary percutaneous coronary intervention [7], [8].

However, this epidemiological success has not translated into meaningful improvements in survival once ventricular septal rupture occurs; in-hospital mortality has remained largely unchanged over decades, frequently exceeding 40–50% even in contemporary series [9]. In our cohort, 30-day mortality reached 54.4%, underscoring the persistent lethality of this condition. Despite the relatively small sample size, contemporary surgical series of post-infarction VSR remain inherently limited by the rarity of the disease. As an example, Sumi et al. reported only 49 surgically treated patients over a 15-year period at a specialized Japanese referral centre, illustrating the ongoing challenges of assembling large contemporary cohorts of post-infarction VSR [10].

Ventricular septal rupture severely impairs left ventricular performance by diverting systolic output away from the systemic circulation, leading to an immediate reduction in effective forward flow. Persistent left-to-right shunting induces acute left ventricular volume overload, increased wall stress, elevated filling pressures, and worsening myocardial oxygen demand in an already infarcted myocardium [11].

The resulting pulmonary overcirculation leads to rising pulmonary pressures and progressive pulmonary hypertension, increasing right ventricular afterload and precipitating right ventricular failure. Adverse ventricular interdependence further compromises left ventricular filling and contractile efficiency, culminating in rapid haemodynamic deterioration and biventricular cardiogenic shock [12], [13].

In our cohort, longer delays to surgical repair were associated with increased mortality. This observation appears to contrast with several contemporary series reporting improved outcomes with delayed intervention [14], [15], [16]; however, this discrepancy is likely explained by the markedly prolonged delays observed in our population. Notably, the median time from symptom onset to diagnosis was 8 days, reflecting substantial pre-hospital and referral delays. In the large multicentre study by Ronco et al., the median time from acute myocardial infarction to ventricular septal rupture was 2 days, with a further median interval of 2 days from diagnosis to surgery [17].

By contrast, patients in our cohort underwent surgical repair substantially later, with a median of 15 days from symptom onset and 6 days from hospital admission, demonstrating a prolonged clinical and surgical timeline compared with contemporary series. Because referral source and transfer status were not consistently available in the structured dataset, we could not reliably determine how much of this delay was related to late presentation versus inter-hospital referral patterns. However, these findings likely reflect the complexity of managing post-infarction VSR in a real-world tertiary-care setting.

Importantly, in multivariable logistic regression analysis, post-operative intra-aortic balloon pump (IABP) use emerged as a strong independent protective factor against 30-day mortality (OR 0.014, 95% CI 0.001–0.120; *p* = 0.001). Pre-operative IABP use was also associated with a substantial reduction in mortality, showing a consistent protective trend that narrowly failed to reach conventional statistical significance (OR 0.161, 95% CI 0.016–1.062; *p* = 0.077). Although confounding by indication cannot be excluded, the persistence of this association after multivariable adjustment and the magnitude of effect observed with post-operative support suggest a clinically relevant haemodynamic contribution beyond patient selection alone.

In this setting, IABP support appears to play a crucial modulatory role. By reducing afterload and left-to-right shunt fraction while improving coronary perfusion and systemic haemodynamics, IABP may stabilise patients sufficiently to allow delayed repair under more favourable myocardial conditions [4], [12], [18].

Although randomized trials failed to demonstrate a mortality benefit of IABP in unselected cardiogenic shock populations, these studies systematically excluded patients with mechanical complications of acute myocardial infarction, including VSR [19]. Consequently, their findings should not be directly extrapolated to this unique clinical scenario.

Importantly, contemporary guideline recommendations continue to support the use of IABP in patients with ACS-related mechanical complications. The 2023 ESC Guidelines recommend that IABP should be considered in patients with haemodynamic instability or cardiogenic shock secondary to mechanical complications of ACS (Class IIa, Level C), while the latest ACC/AHA Guidelines consider temporary mechanical circulatory support reasonable as a bridge to definitive treatment in patients with ACS-related mechanical complications (Class IIa, Level B) [20], [21].

In this context, the intra-aortic balloon pump remains a pragmatic, widely accessible, and lower-cost option, representing a feasible haemodynamic support strategy for patients with post-infarction ventricular septal rupture, particularly in resource-limited settings. Although the limitations of our study preclude definitive conclusions, our findings support continued consideration of IABP use in carefully selected patients with mechanical complications of acute myocardial infarction. Our setting also differed from contemporary mechanical-support programs because ECMO was not part of routine institutional service availability during the study period.

Accordingly, IABP represented the principal temporary mechanical support modality in this cohort, a reality that remains relevant for many tertiary referral centres in low- and middle-income countries, where access to advanced mechanical circulatory support technologies such as VA-ECMO and Impella is frequently constrained by financial costs, infrastructure requirements, and the need for specialized expertise. Therefore, our findings may be particularly applicable to healthcare systems facing similar resource and platform limitations.

Taken together, these findings suggest that while moderate surgical delay may be beneficial by allowing myocardial tissue consolidation, excessive postponement exposes patients to prolonged haemodynamic instability and end-organ dysfunction [22], [23]. In this context, IABP support appears to play a critical modulatory role, mitigating the deleterious effects of prolonged delays and enabling a safer bridge-to-repair strategy in selected patients with post-infarction ventricular septal rupture.

In an era in which intra-aortic balloon pump use has declined following neutral results in unselected cardiogenic shock populations, our study highlights a potential niche role for this device [18], [24], [25]. While contemporary mechanical circulatory support technologies, such as Impella, have demonstrated benefit in selected cardiogenic shock populations, their applicability to mechanical complications and their widespread use in low- and middle-income countries remain limited by cost and availability [24], [26].

Novel therapeutic approaches have been developed, including percutaneous treatment in selected cases. Transcatheter closure techniques have emerged as an alternative strategy to mitigate the high perioperative risk and mortality associated with surgical repair of post-infarction ventricular septal rupture [27], [28]. Device selection remains highly variable and is influenced by operator experience, device availability, and anatomical characteristics of the defect, including size, location, and morphological complexity.

This study should not be interpreted as demonstrating a causal benefit of IABP. Rather, it describes a real-world surgical cohort from a delayed-referral tertiary center in which patients classified as receiving perioperative IABP—particularly in the post-operative category—had lower observed 30-day mortality. Given the small sample size, missing data, possible exposure misclassification, and the risk of confounding by indication, these findings are best understood as hypothesis-generating.

## 5 Limitations

This study has important limitations. Its retrospective, single-centre design is inherently subject to selection bias and residual confounding, precluding causal inference. The relatively small sample size, although reflective of the rarity of post-infarction ventricular septal rupture, limits statistical power, particularly in multivariable analyses. Therefore, adjusted associations should be interpreted as exploratory rather than causal.

Delayed presentation and referral to a tertiary centre were prominent features of this cohort. While this reflects real-world practice in many healthcare systems, it limits the generalisability of our findings to settings with earlier diagnosis and expedited surgical pathways.

The observed association between intra-aortic balloon pump (IABP) use and reduced mortality should be interpreted cautiously, as confounding by indication cannot be fully excluded.

Because post-discharge follow-up was limited and no deaths occurred after hospital discharge up to 30 days, 30-day mortality was reported, which coincided with in-hospital mortality. No additional deaths were observed beyond 30 days; therefore, time-to-event survival analyses were not performed.

Also, the structured source dataset classified IABP only as no IABP, pre-operative IABP, or post-operative IABP and did not permit reliable reconstruction of exclusive versus overlapping perioperative use.

An additional limitation is that advanced mechanical circulatory support devices, such as VA-ECMO and Impella, were not available during the study period and therefore were not used in this cohort. Consequently, our findings reflect a real-world practice setting in which intra-aortic balloon pump support represented the principal temporary mechanical circulatory support strategy.

Finally, the current dataset was restricted to surgically treated patients and did not provide a complete institutional denominator for all post-infarction VSR cases, including patients who died before surgery, were treated conservatively, or underwent definitive transcatheter-only closure.

## 6 Conclusions

In real-world settings characterised by delayed diagnosis and referral, intra-aortic balloon pump support was associated with lower observed 30-day mortality with post-infarction ventricular septal rupture, particularly when used in the peri-operative period. Despite advances in reperfusion and surgical techniques, post-infarction VSR remains a highly lethal condition. Our findings support a pragmatic role for IABP as a bridge-to-repair strategy in contemporary acute cardiovascular care.

## CRediT authorship contribution statement

**Italo Menezes Ferreira:** Writing – review & editing, Writing – original draft, Supervision, Project administration, Methodology, Data curation, Conceptualization. **Mariana Ayaka Yamashita:** Writing – review & editing, Writing – original draft, Data curation. **Marina Carvalho Giannini:** Writing – review & editing, Writing – original draft. **Gabriel Martins Pistori:** Writing – review & editing, Writing – original draft, Data curation. **Vinicius de Amorim e Jorge:** Writing – original draft, Data curation. **Alex Cesar Lacuna:** Writing – original draft, Formal analysis. **Laíz Teixeira Pontes:** Supervision, Data curation. **Raphael Paris Rosan:** Writing – review & editing, Writing – original draft, Supervision. **Antônio Carlos Mugayar Bianco:** Validation, Supervision. **Mario Issa:** Validation, Supervision. **Kleber Franchini:** Project administration, Methodology, Formal analysis. **Fausto Feres:** Writing – original draft, Project administration. **Louis Nakayama Ohe:** Writing – original draft, Validation, Supervision, Investigation.

## Ethical statement

This retrospective cohort study was approved by the Institutional Ethics Committee of the Instituto Dante Pazzanese de Cardiologia, São Paulo, Brazil. Due to the retrospective nature of the study and the use of anonymized clinical data, the requirement for informed consent was waived. The study was conducted in accordance with the ethical principles of the Declaration of Helsinki.

## Funding

This study did not receive any specific grant from funding agencies in the public, commercial, or not-for-profit sectors.

## Declaration of competing interest

The authors declare that they have no conflicts of interest.

## Data Availability

The data underlying this article are available from the corresponding author upon reasonable request.
