# Supplementary material for: Intra-aortic balloon pump support and early mortality after surgical repair of post-infarction ventricular septal rupture: A 24-year real-world cohort
Source: Am Heart J Plus. 2026 Aug 20;70:100863. doi: 10.1016/j.ahjo.2026.100863 (PMC13543087; doi:10.1016/j.ahjo.2026.100863)
Supplement: Supplementary file 1 [file mmc2.docx]

**USO DO BALÃO INTRA AÓRTICO EM PACIENTES COM COMUNICAÇÃO INTERVENTRICULAR PÓS INFARTO AGUDO DO MIOCÁRDIO**

**ANÁLISE ESTATÍSTICA DOS DADOS**

**Outubro de 2025**

**Pesquisadora: Dra Mariana Yamashita**

**Estatística:** Juliana Luz Passos Argenton (CONRE 9822-A)

1. **Objetivo**

O objetivo deste relatório estatístico é avaliar os fatores relacionados ao óbito em pacientes com diagnóstico de comunicação interventricular secundária ao infarto agudo do miocárdio.

2. **Metodologia Estatística**

As análises estatísticas foram realizadas no software R, versão 4.2.2. Foi realizada análise exploratória de dados através do cálculo de frequência absoluta e relativa, média, desvio padrão, mediana, primeiro e terceiro quartil. Os grupos de óbito e sobreviventes foram comparados através do teste Qui-Quadrado ou Mann-Whitney. O modelo de regressão logística com seleção de variáveis stepwise foi usado para encontrar o conjunto de fatores que mais explicam a chance de óbito. O nível de significância adotado foi de 5% (p < 0,05).

3. **Resultados**

Foram incluídos 59 pacientes com diagnóstico de comunicação interventricular (CIV) secundária ao infarto agudo do miocárdio (IAM). As informações apresentadas a seguir descrevem o perfil clínico e laboratorial da amostra, bem como os intervalos de tempo relacionados à evolução clínica até a correção cirúrgica.

A Tabela 1 apresenta as variáveis contínuas avaliadas. Observa-se que a idade média dos pacientes foi de 65,3 anos (DP = 10,2), com mediana de 67 anos. A creatinina apresentou média de 1,39 mg/dL (DP = 0,82). A FEVE apresentou média de 0,43 (DP = 0,10).

O intervalo médio entre a dor e a admissão hospitalar foi de 26,8 dias (DP = 47,4), com mediana de 8 dias. O tempo médio entre a dor e a cirurgia foi de 39,1 dias (DP = 57,7), com mediana de 15 dias, enquanto o tempo entre a admissão e a cirurgia apresentou média de 11,2 dias (DP = 30,6) e mediana de 6,00 dias.

Entre as variáveis laboratoriais, o lactato na admissão teve média de 2,02 mmol/L (DP = 2,36), com diferença média pós-operatória (pós-adm) de -0,66 mmol/L (DP = 2,43). O BNP de admissão apresentou média de 7564 pg/mL (DP = 9066). Ressalta-se que essas variáveis laboratoriais apresentaram número reduzido de observações, variando entre 14 e 16 casos disponíveis.

A Tabela 2 descreve as variáveis categóricas. A amostra foi composta 57,6% de homens e 57,6% de tabagistas. A maioria dos pacientes apresentava hipertensão arterial sistêmica (71,2%), seguida por dislipidemia (50,8%) e diabetes mellitus (35,6%). Doença arterial coronariana prévia foi observada em 74,6% dos casos, e o uso prévio de AAS foi relatado em 59,3%.

O balão intra-aórtico (BIA) foi utilizado em 64,4% dos pacientes, sendo 33,9% de forma pré-operatória e 30,5% no pós-operatório. Hemodiálise foi necessária em 17,2% dos casos, e o uso de cateter de Swan-Ganz foi documentado em 54,5% dos pacientes avaliados.

Em relação aos desfechos, observou-se mortalidade hospitalar em 54,4% dos pacientes com dados disponíveis (N=57). A sobrevida em 30 e 180 dias foi de 53,6%. O tempo entre a dor e a cirurgia foi superior a 10 dias em 66% dos pacientes, enquanto 34% foram operados dentro dos primeiros 10 dias.

A Tabela 3 apresenta a distribuição das artérias coronárias envolvidas nos casos de CIV pós-IAM. A DA foi o vaso mais frequentemente acometido, presente em 62% dos pacientes, seguida pela CD, envolvida em 53%. A CX foi menos frequente, observada isoladamente em 11% dos casos. Combinações de acometimento entre DA e CD foram observadas em 14,5% dos pacientes, enquanto envolvimentos triplos (DA, CX e CD) foram raros (1,8%).

**Tabela 1 –** Características quantitativas da amostra

| **Variável** | **Média (D.P.)** | **Mediana[Q1;Q3]** | **N total** |
| --- | --- | --- | --- |
| Tempo entre dor e adm | 26.8 (47.4) | 8.00 [1.00;26.2] | 54 |
| Idade | 65.3 (10.2) | 67.0 [57.5;72.0] | 59 |
| Creatinina | 1.39 (0.82) | 1.00 [1.00;1.80] | 58 |
| FEVE | 0.43 (0.10) | 0.41 [0.35;0.47] | 56 |
| Lactato na adm | 2.02 (2.36) | 1.40 [0.85;2.60] | 16 |
| Dif Lactato (pós-adm) | -0.66 (2.43) | -0.25 [-1.30;0.08] | 14 |
| BNP na adm | 7564 (9066) | 4015 [0.00;13400] | 16 |
| Tempo entre dor e cirurgia | 39.1 (57.7) | 15.0 [7.00;37.0] | 53 |
| Tempo entre adm e cirurgia | 11.2 (30.6) | 6.00 [2.00;8.00] | 58 |

A Tabela 4 apresenta a comparação das variáveis contínuas entre os grupos de pacientes que evoluíram com e sem óbito. As variáveis foram descritas por média (desvio-padrão) e mediana [Q1;Q3], e as comparações entre grupos foram realizadas através do teste de Mann-Whitney.

Observou-se que a idade foi significativamente menor entre os pacientes que evoluíram com óbito em comparação aos sobreviventes (média de 61,9 vs. 70,1 anos; p=0,002). Também houve diferença significativa no tempo entre dor e cirurgia, que foi maior entre os pacientes que faleceram em relação aos não óbitos (média de 52,4 vs. 23,3 dias; p = 0,015). O tempo entre admissão e cirurgia também diferiu entre os grupos, sendo maior nos óbitos (p = 0,006).

O BNP na admissão mostrou valores mais elevados entre os pacientes que não evoluíram com óbito quando comparados aos óbitos (mediana de 14300 vs. 921; p = 0,044). As demais variáveis, como creatinina, FEVE e lactato, não apresentaram diferenças estatisticamente significativas entre os grupos.

A Tabela 5 apresenta a distribuição das variáveis categóricas de acordo com o desfecho de óbito. As comparações foram realizadas por meio do teste Qui-Quadrado.

Entre as variáveis clínicas e demográficas, não foram observadas diferenças significativas quanto a sexo, tabagismo, hipertensão arterial sistêmica, diabetes mellitus, dislipidemia, doença arterial coronariana prévia, infarto prévio, uso de AAS prévio ou artéria acometida (p > 0,05).

Por outro lado, observou-se associação significativa para uso de balão intra-aórtico (BIA). O grupo sem óbito apresentou maior proporção de uso do dispositivo (p < 0,001), tanto na análise detalhada (pré, pós ou ausência de uso) quanto na forma agrupada (sim/não). A presença de HD também diferiu entre os grupos, sendo mais frequente entre os sobreviventes (32,0% vs. 6,45%; p = 0,017).

As demais variáveis, incluindo o uso de cateter de Swan-Ganz e o tempo entre dor e cirurgia categorizado (≤10 dias ou >10 dias), não mostraram associação significativa com o desfecho.

**Tabela 2 –** Características categóricas e desfechos clínicos da amostra

| **Variável** | **N (%)** | **N total** |
| --- | --- | --- |
| Sexo: |  | 59 |
| Feminino | 25 (42.4%) |  |
| Masculino | 34 (57.6%) |  |
| Tabagista: |  | 59 |
| Não | 25 (42.4%) |  |
| Sim | 34 (57.6%) |  |
| HAS: |  | 59 |
| Não | 17 (28.8%) |  |
| Sim | 42 (71.2%) |  |
| DM: |  | 59 |
| Não | 38 (64.4%) |  |
| Sim | 21 (35.6%) |  |
| DLP: |  | 59 |
| Não | 29 (49.2%) |  |
| Sim | 30 (50.8%) |  |
| DAC: |  | 59 |
| Não | 15 (25.4%) |  |
| Sim | 44 (74.6%) |  |
| IAM: |  | 59 |
| Não | 5 (8.47%) |  |
| Sim | 54 (91.5%) |  |
| AAS prévio: |  | 59 |
| Não | 24 (40.7%) |  |
| Sim | 35 (59.3%) |  |
| BIA: |  | 59 |
| Não | 21 (35.6%) |  |
| pós | 18 (30.5%) |  |
| pré | 20 (33.9%) |  |
| BIA agrupado: |  | 59 |
| Não | 21 (35.6%) |  |
| Sim | 38 (64.4%) |  |
| HD: |  | 58 |
| Não | 48 (82.8%) |  |
| Sim | 10 (17.2%) |  |
| SWAN-GANZ: |  | 22 |
| Não | 10 (45.5%) |  |
| Sim | 12 (54.5%) |  |
| Desfecho: |  | 57 |
| Não óbito | 26 (45.6%) |  |
| Óbito | 31 (54.4%) |  |
| Vivo em 30 dias |  | 56 |
| Não | 26 (46.4%) |  |
| Sim | 30 (53.6%) |  |
| Vivo em 180 dias |  | 56 |
| Não | 26 (46.4%) |  |
| Sim | 30 (53.6%) |  |
| Tempo entre dor e cirurgia: |  | 53 |
| <=10 dias | 18 (34.0%) |  |
| >10 dias | 35 (66.0%) |  |

**Tabela 3 –** Distribuição das combinações de territórios coronarianos acometidos (N=55)

| **Artérias acometidas** | **N das combinações (%)** | **N de pacientes independente da combinação (%)** |
| --- | --- | --- |
| CD | 18 (32.7%) | 29 (53%) |
| CX | 1 (1.82%) | 6 (11%) |
| CX, CD | 1 (1.82%) |  |
| CX,CD | 1 (1.82%) |  |
| DA | 23 (41.8%) | 34 (62%) |
| DA,CD | 8 (14.5%) |  |
| DA,CX | 2 (3.64%) |  |
| DA,CX,CD | 1 (1.82%) |  |

**Tabela 4 –** Comparação das variáveis quantitativas segundo o desfecho de óbito

| **Variável** | **Não Óbito** | | | **Óbito** | | **p-valor** | **N total** |
| --- | --- | --- | --- | --- | --- | --- | --- |
|  | **Média (D.P.)** | **Mediana [Q1;Q3]** | **Média (D.P.)** | | **Mediana [Q1;Q3]** |  |  |
| Tempo entre dor e adm | 9.17 (9.54) | 7.50 [0.75;14.2] | 42.0 (60.5) | | 11.0 [1.00;46.0] | 0.122 | 53 |
| **Idade** | **70.1 (9.32)** | **69.5 [66.0;76.2]** | **61.9 (8.56)** | | **63.0 [54.5;69.0]** | **0.002** | **57** |
| Creatinina | 1.54 (1.03) | 1.00 [1.00;2.00] | 1.26 (0.59) | | 1.00 [1.00;1.00] | 0.274 | 56 |
| FEVE | 0.41 (0.10) | 0.40 [0.35;0.44] | 0.44 (0.11) | | 0.45 [0.36;0.50] | 0.155 | 54 |
| Lactato na adm | 3.50 (3.56) | 2.10 [1.70;3.10] | 1.39 (1.36) | | 1.20 [0.17;2.22] | 0.140 | 15 |
| Dif Lactato (pós-adm) | -1.66 (3.66) | -0.90 [-1.80;0.10] | -0.52 (0.65) | | -0.25 [-1.10;0.00] | 0.825 | 13 |
| **BNP na adm** | **14308 (11630)** | **14300 [5640;19600]** | **4498 (6008)** | | **921 [0.00;8440]** | **0.044** | **16** |
| **Tempo entre dor e cirurgia** | **23.3 (48.8)** | **11.0 [4.50;19.5]** | **52.4 (62.5)** | | **28.0 [10.0;54.0]** | **0.015** | **52** |
| **Tempo entre adm e cirurgia** | **12.9 (45.6)** | **3.00 [1.00;6.00]** | **10.1 (10.4)** | | **7.00 [5.00;12.5]** | **0.006** | **56** |

A Tabela 6 apresenta os resultados do modelo final de regressão logística binária obtido por seleção stepwise, incluindo as variáveis que permaneceram significativamente associadas ao desfecho de óbito após o ajuste multivariado. Entre as variáveis incluídas inicialmente (com p ≤ 0,20 nas análises univariadas), permaneceram no modelo idade, uso de balão intra-aórtico (BIA) no pós e no pré-operatório.

Observou-se que a idade apresentou associação inversa com o óbito (OR = 0,876; p = 0,012), indicando que, a cada aumento de um ano na idade, a chance de óbito diminuiu em aproximadamente 12%.

O uso de BIA pós-operatório apresentou associação com menor probabilidade de óbito (OR = 0,014; p = 0,001), sugerindo um efeito protetor do dispositivo quando utilizado após a cirurgia.

**Tabela 5 –** Comparação das variáveis categóricas segundo o desfecho de óbito

| **Variável** | **Não Óbito N (%)** | **Óbito N (%)** | **p-valor** | **N total** |
| --- | --- | --- | --- | --- |
| Sexo: |  |  | 0.959 | 57 |
| Feminino | 12 (46.2%) | 13 (41.9%) |  |  |
| Masculino | 14 (53.8%) | 18 (58.1%) |  |  |
| Tabagista: |  |  | 0.557 | 57 |
| Não | 13 (50.0%) | 12 (38.7%) |  |  |
| Sim | 13 (50.0%) | 19 (61.3%) |  |  |
| HAS: |  |  | 0.418 | 57 |
| Não | 5 (19.2%) | 10 (32.3%) |  |  |
| Sim | 21 (80.8%) | 21 (67.7%) |  |  |
| DM: |  |  | 0.552 | 57 |
| Não | 18 (69.2%) | 18 (58.1%) |  |  |
| Sim | 8 (30.8%) | 13 (41.9%) |  |  |
| DLP: |  |  | 0.664 | 57 |
| Não | 11 (42.3%) | 16 (51.6%) |  |  |
| Sim | 15 (57.7%) | 15 (48.4%) |  |  |
| DAC: |  |  | 0.944 | 57 |
| Não | 7 (26.9%) | 7 (22.6%) |  |  |
| Sim | 19 (73.1%) | 24 (77.4%) |  |  |
| IAM: |  |  | 0.651 | 57 |
| Não | 3 (11.5%) | 2 (6.45%) |  |  |
| Sim | 23 (88.5%) | 29 (93.5%) |  |  |
| AAS prévio: |  |  | 0.103 | 57 |
| Não | 14 (53.8%) | 9 (29.0%) |  |  |
| Sim | 12 (46.2%) | 22 (71.0%) |  |  |
| Artéria acometida |  |  | NC | 53 |
|  | 9 (36.0%) | 7 (25.0%) |  |  |
|  | 0 (0.00%) | 1 (3.57%) |  |  |
|  | 0 (0.00%) | 1 (3.57%) |  |  |
|  | 0 (0.00%) | 1 (3.57%) |  |  |
|  | 11 (44.0%) | 12 (42.9%) |  |  |
|  | 2 (8.00%) | 6 (21.4%) |  |  |
|  | 2 (8.00%) | 0 (0.00%) |  |  |
|  | 1 (4.00%) | 0 (0.00%) |  |  |
| BIA: |  |  | **<0.001** | 57 |
| Não | 2 (7.69%) | 19 (61.3%) |  |  |
| pós | 15 (57.7%) | 2 (6.45%) |  |  |
| pré | 9 (34.6%) | 10 (32.3%) |  |  |
| BIA agrupado: |  |  | **<0.001** | 57 |
| Não | 2 (7.69%) | 19 (61.3%) |  |  |
| Sim | 24 (92.3%) | 12 (38.7%) |  |  |
| HD: |  |  | **0.017** | 56 |
| Não | 17 (68.0%) | 29 (93.5%) |  |  |
| Sim | 8 (32.0%) | 2 (6.45%) |  |  |
| SWAN-GANZ: |  |  | 1.000 | 22 |
| Não | 5 (50.0%) | 5 (41.7%) |  |  |
| Sim | 5 (50.0%) | 7 (58.3%) |  |  |
| Tempo entre dor e cirurgia: |  |  | 0.367 | 52 |
| <=10 dias | 10 (43.5%) | 8 (27.6%) |  |  |
| >10 dias | 13 (56.5%) | 21 (72.4%) |  |  |

NC = não calculado (poucas observações em pelo menos uma das categorias)

**Tabela 6 –** Regressão Logística binária com stepwise

| **Fator** | **OR** | **IC(95%)** | | **p-valor** |
| --- | --- | --- | --- | --- |
| Idade | 0.876 | 0.778 | 0.962 | 0.012 |
| BIApós | 0.014 | 0.001 | 0.120 | 0.001 |
| BIApré | 0.161 | 0.016 | 1.062 | 0.077 |

Foi verificado que as variáveis “vivo em 30 dias” e “vivo em 180 dias” apresentavam exatamente os mesmos pacientes classificados como óbito na variável “desfecho”, diferindo apenas por um caso em que o paciente foi registrado como óbito na coluna “desfecho”, mas as colunas correspondentes a 30 e 180 dias estavam vazias. Além disso, observou-se ausência de preenchimento nessas duas colunas para diversos pacientes classificados como “não óbito” na variável principal. Diante dessa inconsistência e da sobreposição completa entre os grupos de óbito, optou-se por não realizar análises de sobrevida em 30 e 180 dias, uma vez que resultariam em achados idênticos aos obtidos para o desfecho principal e estariam sujeitos a viés devido aos dados faltantes.
